# Fremanezumab blocks CGRP induced dilatation in human cerebral, middle meningeal and abdominal arteries

**DOI:** 10.1186/s10194-018-0905-8

**Published:** 2018-08-14

**Authors:** Lena Ohlsson, Erik Kronvall, Jennifer Stratton, Lars Edvinsson

**Affiliations:** 1 Department of Clinical Sciences, Division of Experimental Vascular Research, Lund University, BMC A13, Sölvegatan 17, SE-223 62 Lund, Sweden; 2 Department of Clinical sciences, Neurosurgery, Skane University Hospital, Lund, Sweden; 3 Teva Biologics R&D, Redwood City, California, USA; 4 Department of Internal Medicine and Neurosurgery, University Hospital, Lund, Sweden

**Keywords:** CGRP, CGRP receptor antagonist, Antibody, Fremanezumab, Human vessels

## Abstract

**Background:**

Fremanezumab (TEV-48125) is a fully humanized anti-calcitonin gene-related peptide (CGRP) monoclonal antibody (mAb) that has shown positive results in the prevention of episodic migraine and chronic migraine. Previous preclinical studies have revealed CGRP antagonistic effects on intracranial arteries (ICA). The aim of the study was to evaluate the in vitro antagonistic effects of fremanezumab on human arteries.

**Methods:**

Arteries were removed in conjunction with neurosurgery (cerebral, CA, and middle meningeal artery, MMA, *n* = 7) or reconstructive abdominal surgery (abdominal artery, AA, *n* = 6). Ring segments of the vessels were mounted in a sensitive myograph, the functional responses of vasoactive intestinal peptide (VIP), substance P and CGRP in increasing concentrations (10^− 10^–10^− 7^ M) were studied using pre-contraction with 30 mM potassium chloride (KCl). The concentrations of fremanezumab or isotype control antibody (66.7 nM, 0.33 μM, 0.67 μM) were given 30 min prior to CGRP administration.

**Results:**

All included arteries responded with a strong stable contraction to the application of 30 mM KCl. During this pre-contraction, CGRP caused a concentration-dependent relaxation which differed slightly in maximum effect (I_max_) between the types of arteries (ICA = 100%; AA 80%). Fremanezumab (66.7 nM) showed a shift in the IC_50_ value of CGRP, but no significant change in I_max_. At higher doses there was also a reduction of I_max_. For AA, the I_max_ decreased from 71% at 66.7 nM, to 4.5% with 0.33 μM of fremanezumab. Isotype control antibody did not modify the responses. There was no effect on concentration-dependent relaxation with VIP with 66.7 nM of fremanezumab or isotype control.

**Conclusion:**

CGRP relaxes pre-contracted human arteries by 80–100%, but with different IC_50_; the potency range was ICA < AA. The antagonistic effect and potency of fremanezumab was similar, suggesting that there are vasodilatory CGRP receptors present in all studied arteries and that the antibody may have effect in all studied vessels.

## Background

Calcitonin gene-related peptide alpha (αCGRP) is a 37-amino acid neuropeptide belonging to the calcitonin family of peptides. It is abundant in the circulation. CGRP is widely distributed throughout the central, peripheral and enteric nervous systems [1] and has strong vasodilatory properties, especially in the peripheral microvasculature [2] and in the cerebral circulation [3]. CGRP increases cAMP in human, feline and rodent vascular smooth muscle cells (VSMC) [4, 5] and is also active in endothelium-denuded cerebral arteries as well as in meningeal arteries [6]. The vasomotor actions of αCGRP are implied in a variety of both physiological and pathological processes, including migraine and other primary headaches as well as cerebral vasospasm after subarachnoid hemorrhage [7].

Monoclonal antibodies against CGRP or its receptor are currently being evaluated for the prevention of episodic migraine and chronic migraine; all available clinical trials have shown positive results [8–11]. However, as with all new medications, care has to be taken in order to evaluate possible risks of adverse events such as stroke, hypertension, and myocardial infarction; one way is to study human vessels [12].

Fremanezumab is a fully humanized monoclonal antibody that potently and selectively binds to both isoforms (α and β) of CGRP and thereby prevents CGRP from binding to the CGRP receptor. Therapeutic monoclonal antibodies to CGRP such as fremanezumab have been developed because of the very high target specificity, long pharmacokinetic half-lives, and markedly low potential for hepatic toxicity [8, 13] and in phase 2 and 3 clinical studies, fremanezumab was shown to be an effective and safe migraine treatment [8, 14, 15]. Recently, Edvinsson et al. explained the long time it has taken from finding CGRP to the new medications like gepants (small molecules) and the monoclonal antibodies against CGRP or against the CGRP receptor. Human cranial arteries have been a method to understand how CGRP blocking agents modify the vascular responses to CGRP [16].

The present study was designed to evaluate the in vitro vascular effects of fremanezumab (TEV-48125, formerly LBR-101) on isolated human cerebral, middle meningeal and abdominal arteries.

## Methods

Human meningeal (*n* = 4) and cerebral cortical (*n* = 3) arteries were removed during neurosurgical operations for intracranial tumors (4 male and 3 female)*.* Human subcutaneous arteries (*n = 6*, female) were obtained from reconstructive abdominal surgery. The study was approved by the human ethics committee of Lund (LU818–01) and written informed consent was given by all vessel donors.

After removal, the vessels were immediately immersed in 4 °C cold cell culture medium (DMEM containing essential nutrients and antibiotics) (Gipco, ThermoFisher Scientific, Waltham, MA USA 02451). The vessels were carefully dissected and transferred to cold sodium buffer solution (NaCl 119 mM, NaHCO_3_ 15 mM, KCl 4.6 mM, CaCl_2_ 1.5 mM, NaH_2_PO_4_ 1.2 mM, MgCl 1.2 mM and glucose 5.6 mM), pH 7.4, and stored in refrigerator at 4°Covernight.

### Substances

CGRP, vasoactive intestinal peptide (VIP) and substance P were purchased from Bio-Techne (Abingdon, UK). Common substances for buffer preparation were purchased from Sigma-Aldrich (St Louis, MO, USA).

### Myography

In order to evaluate the dilatory response to CGRP (10^− 10^ – 10^− 7^ M), VIP (10^− 10^ – 10^− 7^ M) and substance P (10^− 9^ – 10^− 8^ M), in human arteries a Mulvany-Halpern Wire Myograph (Danish Myo Technology A/S, Aarhus, Denmark) was used as has been previously described [17]. Vessel segments (1–2 mm) were placed in a tissue bath in 5 ml sodium buffer solution (NaCl 119 mM, NaHCO_3_ 15 mM, KCl 4.6 mM, CaCl_2_ 1.5 mM, NaH_2_PO_4_ 1.2 mM, MgCl 1.2 mM and glucose 5.6 mM). The solution was kept at 37 °C and was continuously aerated with air with 5% CO_2_ to maintain pH at 7.4. Depending on the thickness of the artery, the vessels were mounted either on pins 200 μm thick or on two 40 μm wires, which were inserted into the vessel lumen. The pins/wires were connected to a force transducer and a micrometer screw. Normalization was performed by successive manipulation of the micrometer screw until the vessel segments were stretched to 90% of the normal internal circumference, which is the diameter they would have if relaxed under a transmural pressure of 100 mmHg [18]. After 30 min of recovery, viability of the vessel segments were determined by replacing the sodium buffer with a 63.5 mM potassium buffer [19] (NaCl 119 mM, NaHCO_3_ 15 mM, KCl 63.5 mM, CaCl_2,_ 1.5 mM, NaH_2_PO_4,_ 1.2 mM, MgCl 1.2 mM, and glucose 5.6 mM); the contractile response was measured as 100% contraction by the respective vessel segment. After observing the contractile response to high potassium twice, tissue baths were washed in the above mentioned sodium buffer. Endothelial viability was then tested by pre-contract the vessels with 30 mM potassium-buffer, and after stabilization, successive concentrations of substance P 10^− 8^ – 10^− 7^ M were added, which, if the endothelium was viable, would induce a transient vasodilation. After this step, the potassium buffer was again washed out and replaced with the above mentioned sodium buffer. The vessels were pre-treated with either fremanezumab antibody or the isotype control antibody (KLH) for 20 min. To test the dilatory response to CGRP, the vessels were pre-contracted with 30 mM potassium-buffer for 10 min followed by dose response of antibody. The concentrations of the antibodies were 10, 50, and 100 μg/ml (66.7 nM, 0.33 μM, 0.67 μM, respectively) in the vessel baths, which was in the same range as used in the chronic migraine clinical study [8, 20]. Contraction forces were recorded through a PowerLab unit (ADInstruments, Chalgrove, UK).

### Statistics

Relaxations are expressed as percentage of the pre-tension induced by 30 mM potassium buffer. All concentration-response curves were analyzed by iterative non-linear regression analysis using GraphPad Prism 7.02 (GraphPad Corp, San Diego, CA, USA). I_max_ is the maximal response developed to the agonist. IC_50_ is the concentration that produced half maximum dilatation. Results are given as mean +/− standard error of the mean (SEM) and n is the number of patients used in the respective group. Statistical analyses were performed with non-parametric Mann-Whitney (two groups) and Kruskal-Wallis followed by Dunn’s multiple comparison test (three or four groups) non-parametric tests. Results were considered as significant if *p* < 0.05.

## Results

Arteries from human subcutaneous and intracranial arteries were found to have functional endothelium as tested in artery segments pre-contracted by performing the experiments in a buffer containing 30 mM potassium. The addition of substance P resulted in a concentration-dependent relaxation of the artery segments. For subcutaneous arteries, 10^− 9^ M substance P dilated pre-contracted arteries by 49.8% ± 22.7 and at 10^− 8^ M by 66.2% ± 21.4 (mean ± SEM). The relaxant responses were at the same concentrations of substance P in meningeal and cortical arteries 17.3% ± 16.2 and 26.1% ± 20.6, respectively.

**Fig. 1:**
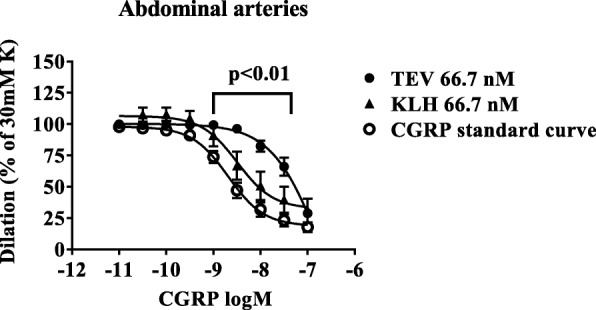
Human abdominal subcutaneous arteries were pre-contracted with 30 mM potassium buffer. The curves show dilatation in response to increasing concentrations of αCGRP 10^− 11^ to 10^− 7^ M in the presence of 66.7 nM of the monoclonal antibody against CGRP (fremanezumab) or an isotype control (KLH). The data show mean ± SEM, *n* = 2–4 for each concentration. CGRP:calcitonin gene-related peptide; K:potassium; TEV:Teva

**Fig. 2:**
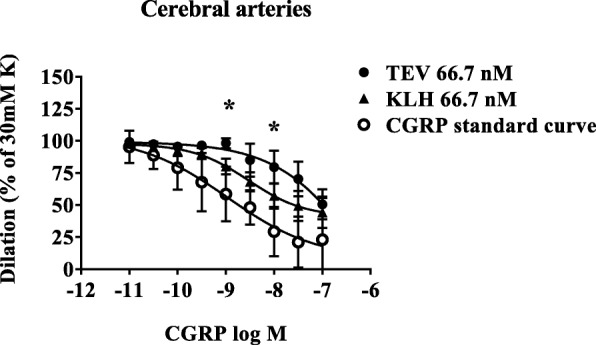
Human intracranial arteries were pre-contracted with 30 mM potassium buffer. The curves show dilatation in response to increasing concentrations of αCGRP 10^− 11^ to 10^− 7^ M in the presence of 66.7 nM of the monoclonal antibody against CGRP (fremanezumab) or an isotype control (KLH). The data show mean ± SEM, *n* = 2–4 for each concentration. CGRP:calcitonin gene-related peptide; K:potassium; TEV:Teva

Following washout with standard buffer solution, the vessels relaxed to baseline and after equilibrium, again the 30 mM potassium buffer was added. In these arteries, CGRP resulted in relaxation by as much as 80–100% of the pre-contraction by increasing concentrations of CGRP (10^− 10^ – 10^− 7^ M) (Figs 1 and 2). We observed no difference in the responses to the endothelial function check at these two time-points.

At the lowest concentration of fremanezumab in the tissue bath (10 mg/L, 66.7 nM), the results showed that the I_max_ was the same as in the control, but the IC_50_ was increased (a shift to the right) for all three types of arteries. In addition, no effect was seen by fremanezumab per se (neither dilatation nor contraction).

At the higher dose of fremanezumab, a delayed dilatation of abdominal arteries was observed with concentrations > 1 nM CGRP. With 100 nM fremanezumab and control, the I_max_ was reduced and the IC_50_ increased (Table 1, Fig. 1). After pre-treatment with 50 or 100 μg/mL (0.33 or 0.67 μM) fremanezumab or isotype control, there was no dilatation with any concentration of CGRP (tested up to 0.1 μM, data not shown). Due to the similar effect in this assay with the isotype control, the effect of these higher antibody concentrations on dilatation cannot be attributed to the sequestration of CGRP.

**Table 1:** Relaxant responses to CGRP in the presence or absence of 66.7 nM fremanezumab or its isotype control

	Abdominal arteries		Intracranial arteries	
	I_max_ (%)	IC_50_ (nM)	I_max_ (%)	IC_50_ (nM)
CGRP control curve	82 ± 4	2.1 ± 0.1	78 ± 22	1.1 ± 0.5
+ fremanezumab 66.7 nM	71 ± 3	182 ± 1.0	49 ± 11	752 ± 9
+ isotype control 66.7 nM	71 ± 9	3.2 ± 0.2	55 ± 12	2.9 ± 0.4

CGRP: calcitonin gene-related peptide, IC_50_: concentration of CGRP in nM that produced half the maximum dilatation, I_max_: maximum dilatation induced by CGRP when the pre-contraction by 30 mM potassium buffer is set to 100%

Values are mean ± SEM

Cerebral and middle meningeal arteries responded in a similar manner with a significantly delayed dilatation in the presence of 10 μg/mL (66.7 nM) fremanezumab antibody (Fig. 2, Table 1). There was a shift in IC_50_ towards the right indicating change in I_max_. Result also showed no relaxation by fremanezumab or the isotype antibody control per se in ICA (data not shown). Addition of 50 μg/mL (0.33 μM) fremanezumab or its isotype control inhibited the CGRP-induced dilatation completely. Therefore, no higher concentration was tested.

**Fig. 3:**
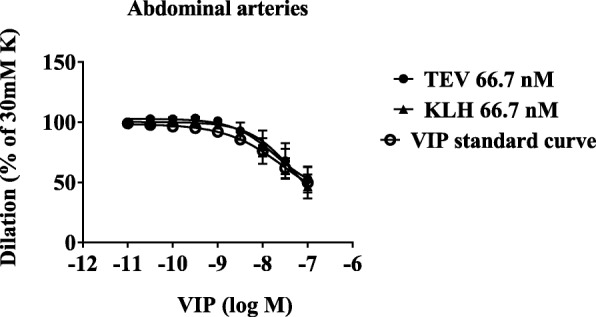
Human abdominal subcutaneous arteries were pre-contracted with 30 mM potassium buffer. The curves show dilatation in response to increasing concentrations of VIP 10^− 11^ to 10^− 7^ M in the presence of 66.7 nM of the monoclonal antibody against CGRP (fremanezumab) or an isotype control (KLH). The data show mean ± SEM, *n* = 2–4 for each concentration. K:potassium; TEV:Teva; VIP:vasoactive intestinal peptide

**Fig. 4:**
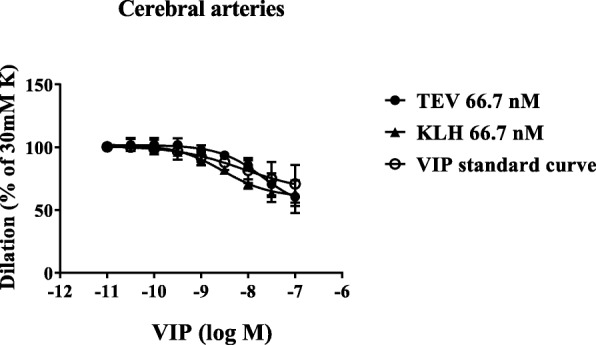
Human intracranial arteries were pre-contracted with 30 mM potassium buffer. The curves show dilatation in response to increasing concentrations of VIP 10^− 11^ to 10^− 7^ M in the presence of 66.7 nM of the monoclonal antibody against CGRP (fremanezumab) or an isotype control (KLH). The data show mean ± SEM, *n* = 2–4 for each concentration. K:potassium; TEV:Teva; VIP:vasoactive intestinal peptide

In abdominal and intracranial arteries, the study tested whether the antibodies had any effect on vasodilatation induced by VIP (Figs. 3 and 4). Neither fremanezumab nor the isotype control antibody had any effect on the VIP-induced dilatation of the cerebral arteries at the doses of 66.7 nM (Figs. 3 and 4) or 0.33 μM (data not shown).

## Discussion

The present study shows that the monoclonal antibody against CGRP, fremanezumab, effectively and selectively blocks the vasomotor responses to CGRP in preparations of human intracranial and peripheral arteries.

Migraine is a highly prevalent disorder characterized by attacks of headache and associated symptoms such as nausea, photophobia, and phonophobia. Most individuals with migraine have episodic migraine, but some have a more severe form known as chronic migraine. The burden of episodic or chronic migraine on the person with migraine, their family, and society at large is substantial [21].

CGRP, a neuropeptide with two isoforms (αCGRP and βCGRP), is believed to be crucial to the pathophysiology of migraine and is therefore an attractive target in the development of migraine preventive strategies. CGRP seems to be involved in peripheral events in migraine (vasodilation, inflammation, and protein extravasation) and is abundantly distributed in the trigeminal ganglion and in the brain [21]. Inhibition of the CGRP pathway has demonstrated efficacy in the acute and preventive treatment of migraine [16, 22, 23]. The first suggestion to use a CGRP receptor antagonist for migraine therapy was made in 1985 [24], however, it has taken more than three decades to reach the clinic [16]. The subsequent testing of such a receptor antagonist or an antagonist antibody on cerebral [25] and middle meningeal arteries [26]. The likely site of action residing outside the blood-brain barrier (BBB) putatively on meningeal vessels or trigeminal ganglion [27, 28].

This study was designed to study in some detail the antagonistic effects of fremanezumab, a fully humanized monoclonal antibody that targets CGRP, on the CGRP-mediated dilatation of human cerebral, meningeal, and abdominal arteries at clinically relevant concentrations. One clear effect was the lack of effect per se of the antibody, thus indicating no tonus by CGRP in these circulations [7]. We observed that the CGRP-induced relaxations in pre-contracted segments behave rather similarly in all three vascular regions and was in concert with previous work (with same IC_50_ and I_max_) [29]. At the lowest concentration of fremanezumab (66.7 nM), CGRP-induced relaxation showed a parallel shift to the right with no change in maximum dilatation and no effect by the isotype control antibody solution. At higher concentrations of fremanezumab, the arterial response to CGRP was markedly reduced or even absent as a sign of strong antagonism. However, the isotype control also had equal effect on arterial relaxation but only at the highest concentration as well, suggesting that some of these data at the higher concentrations may be artefact and is not a contribution of CGRP sequestration. This is commonly seen with high antibody concentrations using in vitro assays and is one of the reasons that we use an isotype control antibody.

It is unknown if fremanezumab may have an effect on several of the receptors belonging to the calcitonin family receptors; future studies may be designed to evaluate also the effects of fremanezumab on the vascular responses to the other calcitonin family member peptides (adrenomedullin, intermedin, amylin and calcitonin). These peptides also activate CGRP receptors so essentially, we are only looking at one component of the receptor action in this in vitro system, which may not represent the normal milieu of peptides occurring in patients.

Previous studies using gepants [29] revealed a competitive antagonistic response with a shift of the concentration-response curve to the right, allowing the calculation of the dissociation constant. The behavior of fremanezumab thus differs from gepants, because the antibody acts as a “sink” in removing CGRP from the myography test system, a likely manner of interaction in patients. The specificity at the 66.7 nM concentration of fremanezumab was verified by unaltered relaxant responses to VIP (a known peptide mediator of VIPR1 and VIPR2 receptors, but has no effect on CGRP receptors) and the contractile effects of 30 mM potassium buffer.

The antibody fremanezumab appears to have a direct functional effect on CGRP-induced relaxation in the tested human arteries with no change in maximum dilatation at the 66, 7 nM concentration. The fremanezumab/CGRP dissociation constant cannot be determined in this experiment due to its extremely high affinity for CGRP, and because the off rate is substantially longer than the time frame of each experiment.

## Conclusion

The results of this study showed that the humanized monoclonal antibody fremanezumab can compete with CGRP to a similar extent in different human arteries. The long half-life of the interaction is relevant in a “chronic” situation or in prophylaxis because a patient will be exposed to the antibody for a longer period of time and will therefore, at some point, be at steady state with respect to the equilibrium constant of the antibody/antigen interaction. The antagonistic effect and potency of fremanezumab was similar in the different arteries studied, suggesting that there are vasodilatory CGRP receptors present in all studied arteries and that the antibody may have effect in all studied vessels.

## Abbreviations

AA: Abdominal artery
BBB: Blood brain barrier
CA: Cerebral artery
CGRP: Calcitonin gene-related peptide
ICA: Intracranial artery
MMA: Middle meningeal artery
VIP: Vasoactive intestinal peptide
VSMC: Vascular smooth muscle cell

## References

[1] Uddman R, Edvinsson L, Ekblad E, Hakanson R, Sundler F (1986). Calcitonin gene-related peptide (CGRP): perivascular distribution and vasodilatory effects. Regul Pept.

[2] Brain SD, Williams TJ, Tippins JR, Morris HR, MacIntyre I (1985). Calcitonin gene-related peptide is a potent vasodilator. Nature.

[3] Uddman R, Edvinsson L, Ekman R, Kingman T, McCulloch J (1985). Innervation of the feline cerebral vasculature by nerve fibers containing calcitonin gene-related peptide: trigeminal origin and co-existence with substance P. Neurosci Lett.

[4] Crossman DC, Dashwood MR, Brain SD, McEwan J, Pearson JD (1990). Action of calcitonin gene-related peptide upon bovine vascular endothelial and smooth muscle cells grown in isolation and co-culture. Br J Pharmacol.

[5] Edvinsson L, Fredholm BB, Hamel E, Jansen I, Verrecchia C (1985). Perivascular peptides relax cerebral arteries concomitant with stimulation of cyclic adenosine monophosphate accumulation or release of an endothelium-derived relaxing factor in the cat. Neurosci Lett.

[6] Edvinsson L, Mulder H, Goadsby PJ, Uddman R (1998). Calcitonin gene-related peptide and nitric oxide in the trigeminal ganglion: cerebral vasodilatation from trigeminal nerve stimulation involves mainly calcitonin gene-related peptide. J Auton Nerv Syst.

[7] Goadsby PJ, Holland PR, Martins-Oliveira M, Hoffmann J, Schankin C, Akerman S (2017). Pathophysiology of migraine: a disorder of sensory processing. Physiol Rev.

[8] Bigal ME, Edvinsson L, Rapoport AM, Lipton RB, Spierings EL, Diener HC (2015). Safety, tolerability, and efficacy of TEV-48125 for preventive treatment of chronic migraine: a multicentre, randomised, double-blind, placebo-controlled, phase 2b study. Lancet Neurol.

[9] Dodick DW, Goadsby PJ, Spierings EL, Scherer JC, Sweeney SP, Grayzel DS (2014). Safety and efficacy of LY2951742, a monoclonal antibody to calcitonin gene-related peptide, for the prevention of migraine: a phase 2, randomised, double-blind, placebo-controlled study. Lancet Neurol.

[10] Goadsby PJ, Sprenger T (2010). Current practice and future directions in the prevention and acute management of migraine. Lancet Neurol.

[11] Sun H, Dodick DW, Silberstein S, Goadsby PJ, Reuter U, Ashina M (2016). Safety and efficacy of AMG 334 for prevention of episodic migraine: a randomised, double-blind, placebo-controlled, phase 2 trial. Lancet Neurol.

[12] MaassenVanDenBrink A, Meijer J, Villalon CM, Ferrari MD (2016). Wiping out CGRP: potential cardiovascular risks. Trends Pharmacol Sci.

[13] Schuster NM, Rapoport AM (2016). New strategies for the treatment and prevention of primary headache disorders. Nat Rev Neurol.

[14] Bigal ME, Dodick DW, Rapoport AM, Silberstein SD, Ma Y, Yang R (2015). Safety, tolerability, and efficacy of TEV-48125 for preventive treatment of high-frequency episodic migraine: a multicentre, randomised, double-blind, placebo-controlled, phase 2b study. Lancet Neurol.

[15] Silberstein SD, Dodick DW, Bigal ME, Yeung PP, Goadsby PJ, Blankenbiller T (2017). Fremanezumab for the preventive treatment of chronic migraine. N Engl J Med.

[16] Edvinsson L, Haanes KA, Warfvinge K, Krause DN (2018). CGRP as the target of new migraine therapies - successful translation from bench to clinic. Nat Rev Neurol.

[17] Edvinsson L, Ekman R, Goadsby PJ (2010). Measurement of vasoactive neuropeptides in biological materials: problems and pitfalls from 30 years of experience and novel future approaches. Cephalalgia.

[18] Mulvany MJ, Halpern W (1977). Contractile properties of small arterial resistance vessels in spontaneously hypertensive and normotensive rats. Circ Res.

[19] Hogestatt ED, Andersson KE, Edvinsson L (1983). Mechanical properties of rat cerebral arteries as studied by a sensitive device for recording of mechanical activity in isolated small blood vessels. Acta Physiol Scand.

[20] Cohen-Barak O, Weiss S, Rasamoelisolo M, Faulhaber N, Yeung PP, Loupe PS et al (2018) A phase 1 study to assess the pharmacokinetics, safety, and tolerability of fremanezumab doses (225 mg, 675 mg and 900 mg) in Japanese and Caucasian healthy subjects. Cephalalgia:333102418771376. 10.1177/0333102418771376 10.1177/0333102418771376 29667896

[21] Warfvinge K, Edvinsson L (2017) Distribution of CGRP and CGRP receptor components in the rat brain. Cephalalgia:333102417728873. 10.1177/0333102417728873 10.1177/0333102417728873 28856910

[22] Edvinsson L, Linde M (2010). New drugs in migraine treatment and prophylaxis: telcagepant and topiramate. Lancet.

[23] Ho TW, Edvinsson L, Goadsby PJ (2010). CGRP and its receptors provide new insights into migraine pathophysiology. Nat Rev Neurol.

[24] Edvinsson L (1985). Functional-role of perivascular peptides in the control of cerebral-circulation. Trends Neurosci.

[25] Edvinsson L (2007). Novel migraine therapy with calcitonin gene-regulated peptide receptor antagonists. Expert Opin Ther Targets.

[26] Juhl L, Edvinsson L, Olesen J, Jansen-Olesen I (2007). Effect of two novel CGRP-binding compounds in a closed cranial window rat model. Eur J Pharmacol.

[27] Eftekhari S, Salvatore CA, Johansson S, Chen TB, Zeng Z, Edvinsson L (2014) Localization of CGRP, CGRP receptor, PACAP and glutamate in trigeminal ganglion. Relation to the blood-brain barrier. Brain Res. 10.1016/j.brainres.2014.11.031 10.1016/j.brainres.2014.11.031 25463029

[28] Lundblad C, Haanes KA, Grande G, Edvinsson L (2015). Experimental inflammation following dural application of complete Freund’s adjuvant or inflammatory soup does not alter brain and trigeminal microvascular passage. J Headache Pain.

[29] Edvinsson L, Chan KY, Eftekhari S, Nilsson E, de Vries R, Saveland H (2010). Effect of the calcitonin gene-related peptide (CGRP) receptor antagonist telcagepant in human cranial arteries. Cephalalgia.

